# Nonlinear viscoelastic models improve characterisation of 6 DOF intervertebral disc load response at low strain rates

**DOI:** 10.1177/09544119251411015

**Published:** 2026-01-25

**Authors:** Samantha Hayward, Patrick S. Keogh, Anthony W. Miles, Sabina Gheduzzi

**Affiliations:** 1 Department of Mechanical Engineering, University of Bath, Claverton Down, Bath, UK

**Keywords:** spine biomechanics, intervertebral disc, viscoelastic, in vitro, biomechanical testing/analysis, mechanical characterisation, methodologies

## Abstract

The viscoelastic characteristics of the intervertebral disc (IVD) govern spinal response to applied dynamic loading which is important in understanding how the spine responds to loads experienced in everyday activity. The common method of reporting experimental load response data in terms of linear stiffnesses represents a significant oversimplification of this behaviour. This study presents a method yielding substantially increased accuracy for principal direction load-displacement response of porcine lumbar spine segments. It compares quality of fit to experimental data of nonlinear viscoelastic models and the typical linear stiffness method. Experimental load response data were recorded from six porcine lumbar spine segments tested under 6 DOF cyclic displacement control at low strain rates (0.1 Hz). Model spring and damper coefficients were determined using an optimisation procedure to minimise the differences between model and experimental load response vectors in each axis. Experimental hysteresis area cannot be reproduced using the linear method but was replicated to within 17% by nonlinear viscoelastic models. Fit quality was substantially improved by nonlinear models compared to the linear stiffness model, with RMSE reduced by 60%. Results indicate that three-element nonlinear viscoelastic models are well-suited for characterisation of principal direction load response to cyclic loading, replicating key features.

## Graphical abstract

**Graphical abstract:**
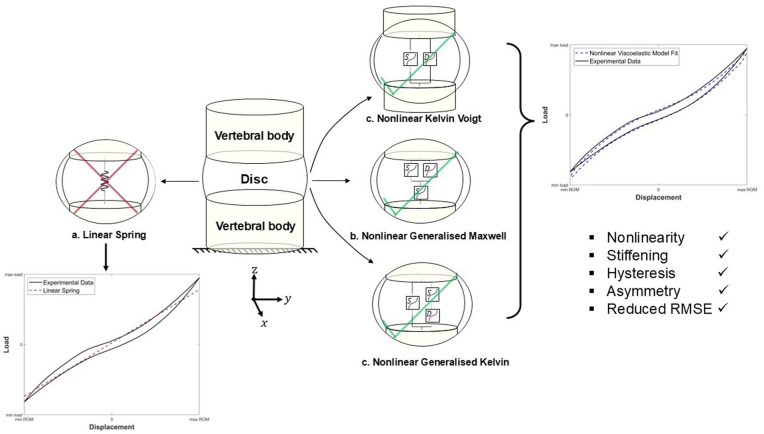

## Introduction

The most common method for reporting data from cyclic tests on spinal segments is the linear stiffness method which represents disc response as a linear elastic spring (Table 1a).^1–8^ Linear regression on experimental load-displacement curves determines the line of best fit, with gradient reported as the stiffness.^1^ This method is favoured for speed, simplicity, repeatability and its usefulness in the formulation of the 6 × 6 linear stiffness or flexibility matrices,^4,8^ but it disregards important features since the tissue is assumed to have linear elastic mechanical characteristics.

**Table 1.** The four confiurations used to model the load-displacement behaviour in each of the six principal axes: (a) a simple linear spring (LS); (b) a nonlinear Kelvin-Voigt (n-KV) element, (c) a nonlinear Generalised Maxwell body of order one (n-GM), and (d) a nonlinear Generalised Kelvin body of order one (n-GK). Constitutive equations are indicated.

Name	Model configuration	Constitutive equations
a. Linear spring (LS)	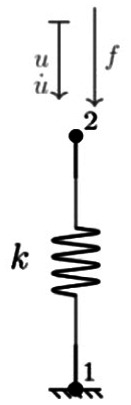	$u=u_{21}$ f=ku
b. Nonlinear Kelvin-Voigt (n-KV)	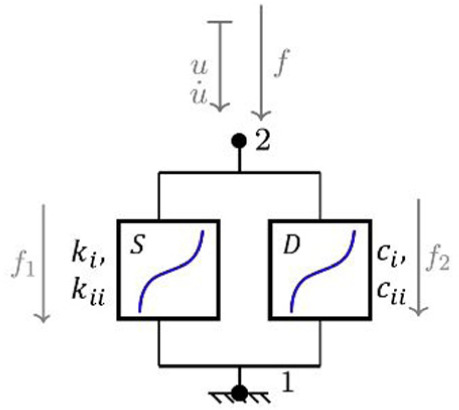	$u=u_{21}$ $\overset{\cdot }{u}={\overset{\cdot }{u}}_{21}$ $f=f_{1}+f_{2}$ $f_{1}=k_{i}u+k_{\mathrm{ii}}u^{3}$ $f_{2}=c_{i}\overset{\cdot }{u}+c_{\mathrm{ii}}{\overset{\cdot }{u}}^{3}$
c. Nonlinear Generalised Maxwell (n-GM)	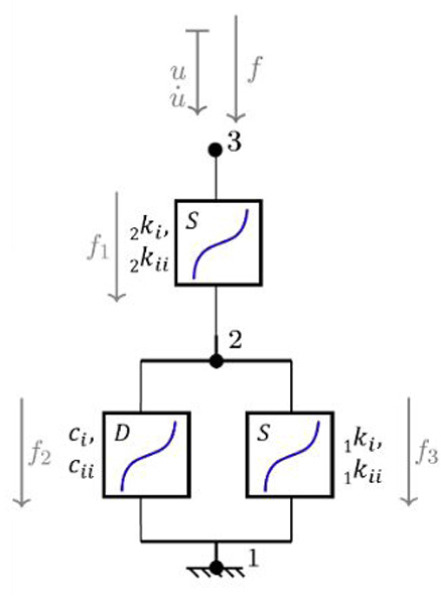	$u=u_{21}+u_{32}$ $f=f_{1}=f_{2}+f_{3}$ $f_{1}=$ _2_ $k_{i}u_{32}+$ _2_ $k_{\mathrm{ii}}u_{32}^{3}$ $f_{2}=$ _1_ $k_{i}u_{21}+$ _1_ $k_{\mathrm{ii}}u_{21}^{3}$ $f_{3}=c_{i}{\overset{\cdot }{u}}_{21}+c_{\mathrm{ii}}{\overset{\cdot }{u}}_{21}^{3}$
d. Nonlinear Generalised Kelvin (n-GK)	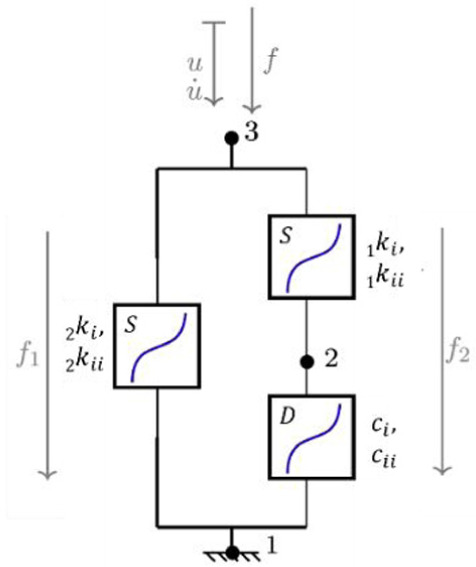	$u=u_{31}=u_{32}+u_{21}$ $f_{1}=$ _2_ $k_{i}u_{31}+$ _2_ $k_{\mathrm{ii}}u_{31}^{3}$ $f_{2}=$ _1_ $k_{i}u_{32}+$ _1_ $k_{\mathrm{ii}}u_{32}^{3}=c_{i}{\overset{\cdot }{u}}_{21}+c_{\mathrm{ii}}{\overset{\cdot }{u}}_{21}^{3}$
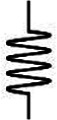 Linear spring	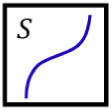 Nonlinear spring	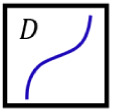 Nonlinear damper

Intervertebral disc (IVD) behaviour is complex and nonlinear in terms of load-displacement response. Data from cyclic testing exhibit nonlinearities including strain stiffening, asymmetry and hysteresis.^9–17^ Asymmetry is common in spinal load-displacement behaviour both in vitro and in vivo, specifically in bending.^18^ Anatomical asymmetry and possible structural weaknesses or defects may lead to compensation resulting in injury or premature degeneration.^19^ Hysteresis yields an area enclosed by load-displacement curves; a measure of energy dissipated per displacement cycle in in vitro experiments. Hysteresis and nonlinear characteristics are correlated with hydration and health of disc tissue and the inner nucleus pulposus (NP), which functions to absorb and dissipate loads during motion.^20^ An effective method of capturing and describing all important characteristics of IVD response is crucial to developing full understanding of spinal response to motion and how tissue health can affect behaviour.

The viscoelastic nature of biological structures is widely accepted,^21–28^ and the IVD in particular exhibits complex viscoelastic behaviour due to its biphasic composition comprising the solid annulus fibrosus (AF) and the fluid-rich NP. Rheological and viscoelastic models are commonly used to represent this type of behaviour, particularly under cyclic or time-dependent loading conditions. In 1991 Tamaki and Panjabi hypothesised that spinal load-displacement behaviour in bending could be described using a cubic nonlinear spring element in series with a linear Kelvin-Voigt element.^29^ In 2003 Izambert et al.^30^ tested the disc in isolation, and using a Kelvin-Voigt element, assessed dynamic axial stiffness over a range of frequencies from 5 to 30 Hz. In 2001 Nicholson et al.^31^ assessed similar viscoelastic models for spine response in an in vivo study. The use of viscoelastic models in multi-axis characterisation of the spine in vitro is exploited in creep, relaxation and impact studies.^21,22,26,28,32–39^, Viscoelastic characteristics have also been included in finite element (FE) models of the IVD validated against in vitro mechanical tests including creep and relaxation.^38,40,41^ However, viscoelastic models have been explored in cyclic testing only for bending^29^ and axial compression.^30,42,43^

In this study, three nonlinear viscoelastic model configurations (Table 1) are investigated and compared to the linear stiffness method: Kelvin-Voigt (n-KV) (Table 1b) representing simultaneous elastic and viscous response, generalised Maxwell body, order one (n-GM) (Table 1c) capturing immediate and delayed elastic response, and generalised Kelvin body, order one (n-GK) (Table 1d) allowing for more flexible representation of hysteresis.

These models have been used in previous studies to simulate creep, stress relaxation and dynamic loading of spinal segments.^28,43,44^ Recent advances in finite element modelling and nonlinear parameter identification have further highlighted the importance of selecting appropriate constitutive models to capture the mechanical behaviour of the IVD under physiological conditions.^45,46^

The present study contributes to this field by validating nonlinear viscoelastic models against experimental data from porcine lumbar spine segments, with the aim of establishing a robust methodology to improve the characterisation of the IVD six degree of freedom (DOF) response at low strain rates. This paper presents the design and results of a method for fully characterising the load-displacement curves of porcine lumbar isolated spinal disc (ISD) specimens using nonlinear viscoelastic models such that the characterising coefficients correspond to specific key features of behaviour.

## Materials and methods

### Specimen preparation and cyclic testing

**Figure 1.**
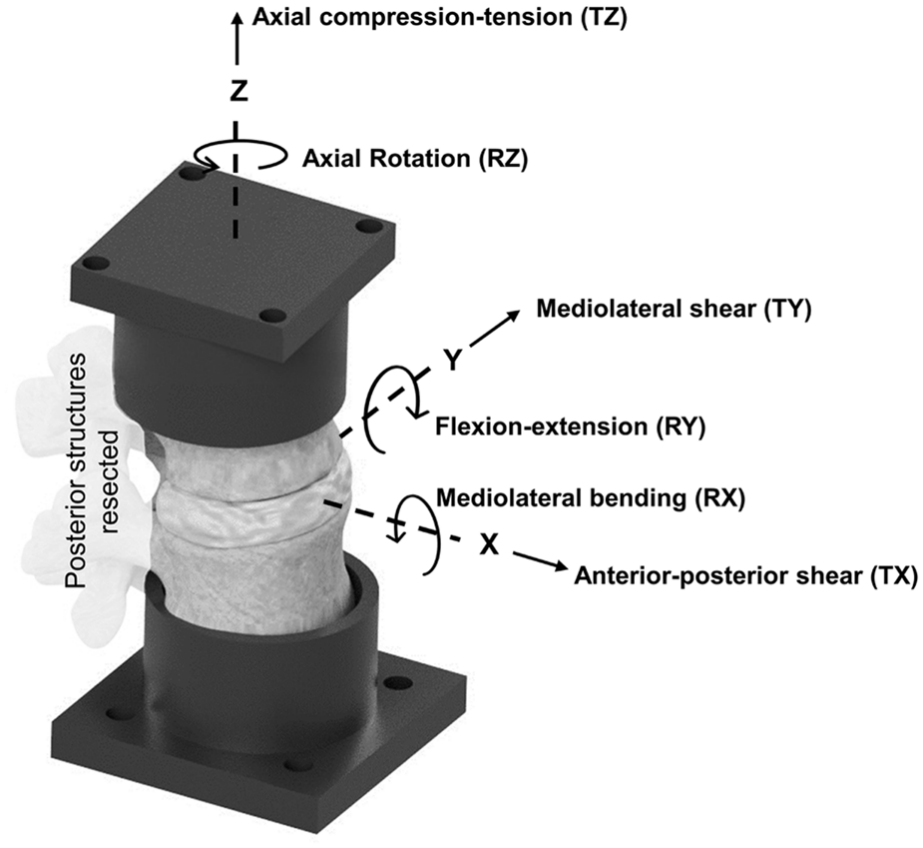
Diagram illustrating the specimen potting and coordinate axes definition along with principal axes of motion.

Three lumbar spinal motion segment specimens from spinal levels L1-L2, L3-L4 and L5-L6 were dissected from each of two fresh porcine spines obtained from a local butcher. This yielded a sample size of six specimens, two from each lumbar level (2 × L1-L2, 2 × L3-L4, 2 × L5-L6). To focus on the viscoelastic behaviour of the IVD unconfounded by the effects of posterior structures such as the facet joints, all posterior structures were resected. This left six isolated spinal disc (ISD) specimens consisting of superior and inferior vertebral bodies, adjoining intervertebral disc, and anterior and posterior longitudinal ligaments (Figure 1). The specimens were sprayed liberally with 0.9% saline-solution, wrapped in saline-soaked tissue, triple-bagged and frozen at −20°C ± 2°C until testing.

Specimen preparation, hydration, potting and alignment was performed according to the method described in.^47^ Before testing, each specimen was thawed for 3 h and potted using Wood’s metal, taking care to keep the disc parallel to the horizontal plane. The exposed portion was sprayed with 0.9% saline solution and wrapped in saline-soaked tissue followed by plastic wrap to minimise dehydration.^3,48^ Using a custom 6-axis spine simulator operating under load control, a 400 N axial preload was applied to each specimen gradually applied using a linear ramp over 15 min to induce creep and initiate fluid redistribution within the disc. This 400 N load was then maintained for 30 min to allow the specimen to reach a near-equilibrium state. The 400 N load was maintained during testing to simulate the axial load on the spine in vivo.

Tests were performed at a room temperature of 20°C ± 2°C and consisted of five cycles of a 0.1 Hz triangular displacement waveform applied in each of the six axes, one at a time in a random order to avoid any effects from order of testing. This relatively low strain rate was chosen to simulate tasks such as gentle stretching, slow flexion and other everyday movements where time-dependent behaviours like viscoelastic relaxation and fluid redistribution within the disc are most pronounced. A triangular displacement waveform was chosen so that specimen behaviour could be evaluated under constant velocity conditions.

Specimens were tested under position control, with displacement applied along a single axis corresponding to one of the six degrees of freedom (DOF). The remaining five DOFs were held stationary during each test to prevent uncontrolled motion and ensure consistent loading conditions. Forces and moments were recorded in all six DOFs.

Displacements and rotations at IVD centre were within a physiologically relevant range for porcine lumbar spine segments, similar to values previously reported.^4,49,50^ Displacement amplitudes were selected to ensure that structural damage due to excessive strain was avoided during mechanical testing. The amplitudes in each axis were: ±1.5 mm in anterior-posterior shear (TX), ±0.75 mm in mediolateral shear (TY), ±0.25 mm in axial compression-tension (TZ), ±4° in mediolateral bending (RX) and flexion-extension (RY), and ±2° in axial torsion (RZ) (Figure 1).

### Data processing

**Figure 2.**
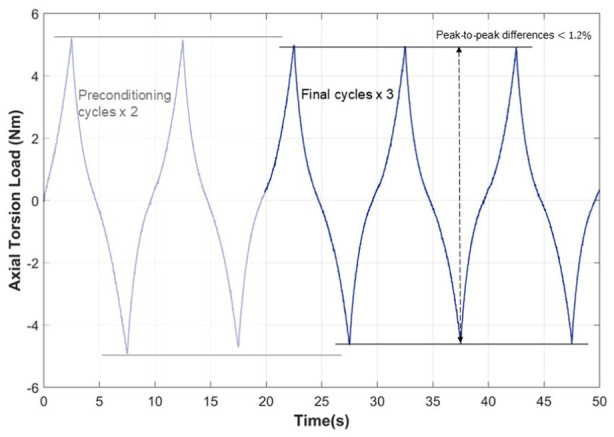
Representative illustration of load-time plot showing all five cycles of loading and indicating the increased variation in the first two cycles which are regarded as mechanical preconditioning

In each principal axis, the first two cycles of each test were treated as mechanical specimen preconditioning and were therefore not included in data analysis as early cycles of loading typically exhibit transient behaviour not representative of the steady-state response.^4,18,51^ Load data from the final three cycles was repeatable – within 3.2% of the load range in each principal direction and peak-to-peak differences between cycles below 1.2% of total load range across all axes (Figure 2). Therefore, these final three cycles were averaged and reproduced to yield three identical cycles of data which formed the optimisation input to the models. This improved the efficiency of the model optimisation by removing minor variations between the three cycles. The averaged data curves were centred in the *y*-axis (load) at zero displacement. This pre-conditioning step ensured that all data were centred around a known point, removing any experimental load offsets and simplifying the application of any initial conditions to the viscoelastic models. These data were used to plot load-displacement curves for the six principal directions.

### Viscoelastic model configurations

The linear method represents IVD response as a linear elastic spring (LS) (Table 1a). Because of its commonplace use, this model was used as the benchmark for goodness of fit comparison. Three further models (Table 1b, c and d) were investigated for suitability in characterising load-displacement behaviour in each principal direction.

A review of relevant literature comprising studies using multi-element viscoelastic configurations to describe intervertebral disc behaviour, suggested that models best suited to characterise IVD response are the Kelvin-Voigt (KV) configuration (Table 1b), a generalised Maxwell body, order one (GM) (Table 1c) and a generalised Kelvin body, order one (GK) (Table 1d).^28,29,52–55^ The solutions of these models reproduce behaviour characterised by stiffening effects at ROM extremes and a central zone of lower stiffness, similar to elastic and neutral zone behaviour seen in spine specimens under applied displacement.^12,56,57^ These three configurations, containing nonlinear sub-elements, are the simplest models which reproduce this behaviour.

These three nonlinear models were compared to the linear elastic spring for goodness of fit provided to experimental data for the six principal directions in each specimen. The generalised constitutive equations for these nonlinear models are presented alongside the model configurations in Table 1.

To adequately describe stiffening and nonlinearity, all elements in the multielement viscoelastic models (Table 1b, c, d) are nonlinear, and are designated by n-KV, n-GM and n-GK, respectively. The state equation for each model element was limited to cubic (Equations (1) and (2)).

Force, F , in each nonlinear spring and damper subject to displacement, x , and velocity, v , are expressed:

(1) $$\begin{array}{c} F_{\mathrm{spring}}=\left\{ \begin{array}{c} K_{\mathrm{ip}}x+K_{\mathrm{iip}}x^{3},x>0\\ K_{\mathrm{in}}x+K_{\mathrm{iin}}x^{3},x<0 \end{array}\right. \end{array}$$

(2) $$F_{\mathrm{damper}}=\left\{ \begin{array}{c} c_{\mathrm{ip}}v+c_{\mathrm{iip}}v^{3},v>0\\ c_{\mathrm{in}}v+c_{\mathrm{iin}}v^{3},v<0 \end{array}\right.$$

where $K_{i},K_{\mathrm{ii}}$ and $c_{i},c_{\mathrm{ii}}$ are linear and cubic stiffness and damping coefficients, respectively.

The pre-conditioning of experimental data to remove any offset in load removed the need for a constant offset term in the state equations. Quadratic terms are not able to maintain the direction of displacement and velocity. Therefore, a cubic polynomial without any quadratic term was chosen to retain information of the direction of velocity and displacement in the state equations. To account for asymmetry specifically in flexion-extension and mediolateral bending, asymmetric models were generated with different sets of stiffness and damping coefficients, $K_{i(p,n)},K_{\mathrm{ii}(p,n)}$ and $c_{i(p,n)},c_{\mathrm{ii}(p,n)}$ , for positive (p) and negative (n) displacements and velocities, respectively. Similar asymmetric models were employed for data in axial compression-extension to account for asymmetric behaviour because of the applied axial preload.

In response to cyclic motions, these nonlinear models reproduce behaviour characterised by a cubic elastic load response governed by the nonlinear spring elements, and hysteresis, arising from energy dissipation from the nonlinear viscous damper.

### Parameter Identification

For each specimen, cyclic displacement was applied in one principal direction at a time, and the resulting load response was recorded across all six degrees of freedom. The displacement vector served as the independent input to the nonlinear viscoelastic model, while the corresponding load vector was the dependent output.

To fit the model parameters, the same cyclic displacement waveforms used in the experiments were applied to the constitutive models. A nonlinear least squares optimisation procedure with trust-region-reflective algorithm in MATLAB (MathWorks, Inc., Natick, MA, USA, version R2020b) iteratively adjusted the stiffness and damping coefficients to minimise the sum of squared errors (SSE) between the entire experimental load vector and the model-predicted load vector. This optimisation was performed on the full hysteresis loop rather than separately on loading and unloading segments, ensuring that the fitted parameters represent the overall cyclic behaviour rather than one phase of the curve. This optimisation was performed separately for each model configuration and each principal direction.

The resulting parameter sets, comprising stiffness and damping coefficients, fully described the model behaviour for each specimen and direction. These parameters were then used to evaluate model performance, including replication of hysteresis area, which was calculated from both experimental data and model predictions.

## Results

**Figure 3.**
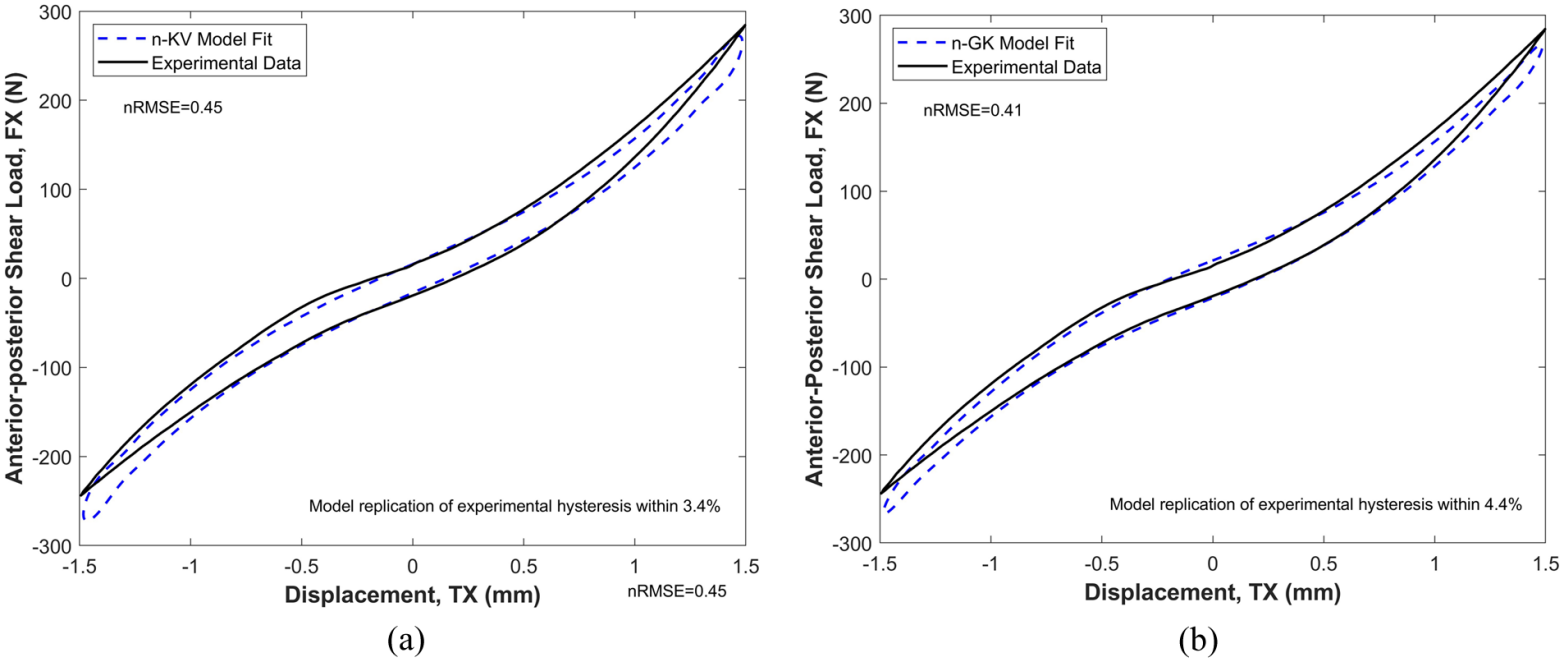
Illustration of the difference in quality of fit provided by the n-KV and n-GK model to experimental data in axial compression-tension. The n-GK model offers the best quality of fit (nRMSE = 0.41) to the experimental data, but the replicated hysteresis area differs from the experimental by 3.6%. The n-KV model replicates the experimental hysteresis area within 3.4% but offers the lowest overall quality of fit (nRMSE = 0.45): (a) Best fit of the Kelvin-Voigt (n-KV) model to experimental data in anterior-posterior shear (TX) and (b) Best fit of the Generalised Kelvin (n-GK) model to experimental data in anterior-posterior shear (TX).

In general, the quality of fit provided by the n-KV model decreases at the extremes of ROMs (Figure 3a). This was improved by the n-GM and n-GK models. An example is shown in Figure 3b for specimen 1 (L1-L2) in anterior-posterior shear. Due to minimal differences in the visual quality of fit of the three-element (n-GM and n-GK) models, only the better of the two – the n-GK model is shown here (Figure 3) in comparison with the n-KV model.

The n-GK and n-GM models consistently provided lower error (RMSE) compared to experimental data than the simpler n-KV model. In anterior-posterior shear, mediolateral shear and axial rotation, the symmetric n-GK model provided the best fit to experimental data across the sample size. In axial compression-extension, mediolateral bending and flexion-extension, the asymmetric n-GM model provided the best fit to experimental data across the sample size.

**Figure 4.**
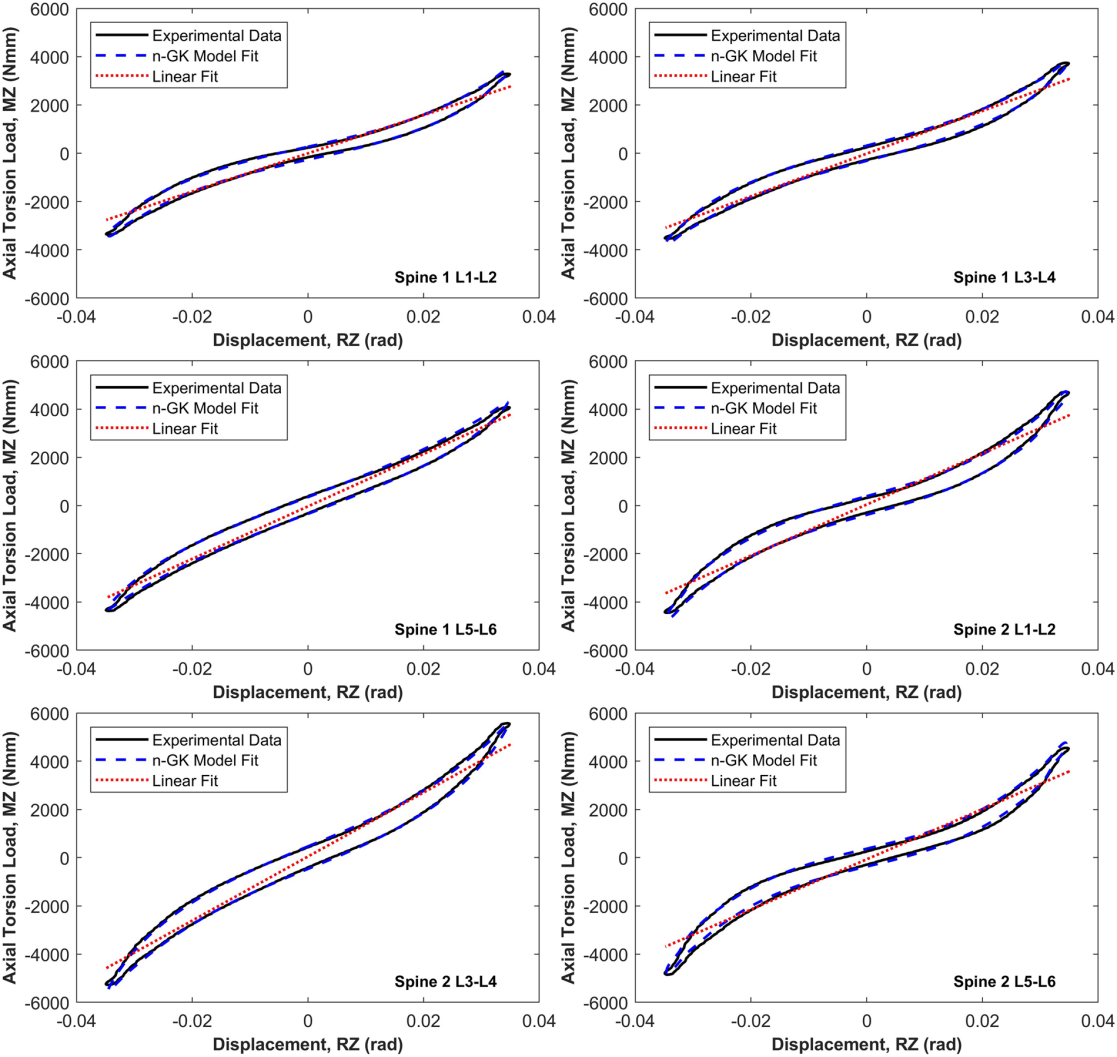
Resulting best fit of the first order Generalised Kelvin (n-GK) model to the axial torsion (RZ) principal element load-displacement behaviour for each of the six specimen tests. The linear model (LS) is also indicated for comparison.

Figure 4 presents the comparison between the Linear Spring (LS) model and the symmetric nonlinear Generalised Kelvin (n-GK) model in axial torsion (RZ) for all six specimens. The n-GK model consistently demonstrated a good fit to the experimental data across all specimens in this direction. Similar quality of fit was observed in anterior-posterior shear (TX) and mediolateral shear (TY), with minimal variation between specimens. Due to the consistency of these results, the corresponding figures for TX and TY are provided in the supplementary material.

In mediolateral bending (RX), the asymmetric nonlinear Generalised Maxwell (n-GM) model also showed strong agreement with experimental data across all specimens. These results are likewise included in the supplementary material to maintain clarity and focus in the main text.

**Figure 5.**
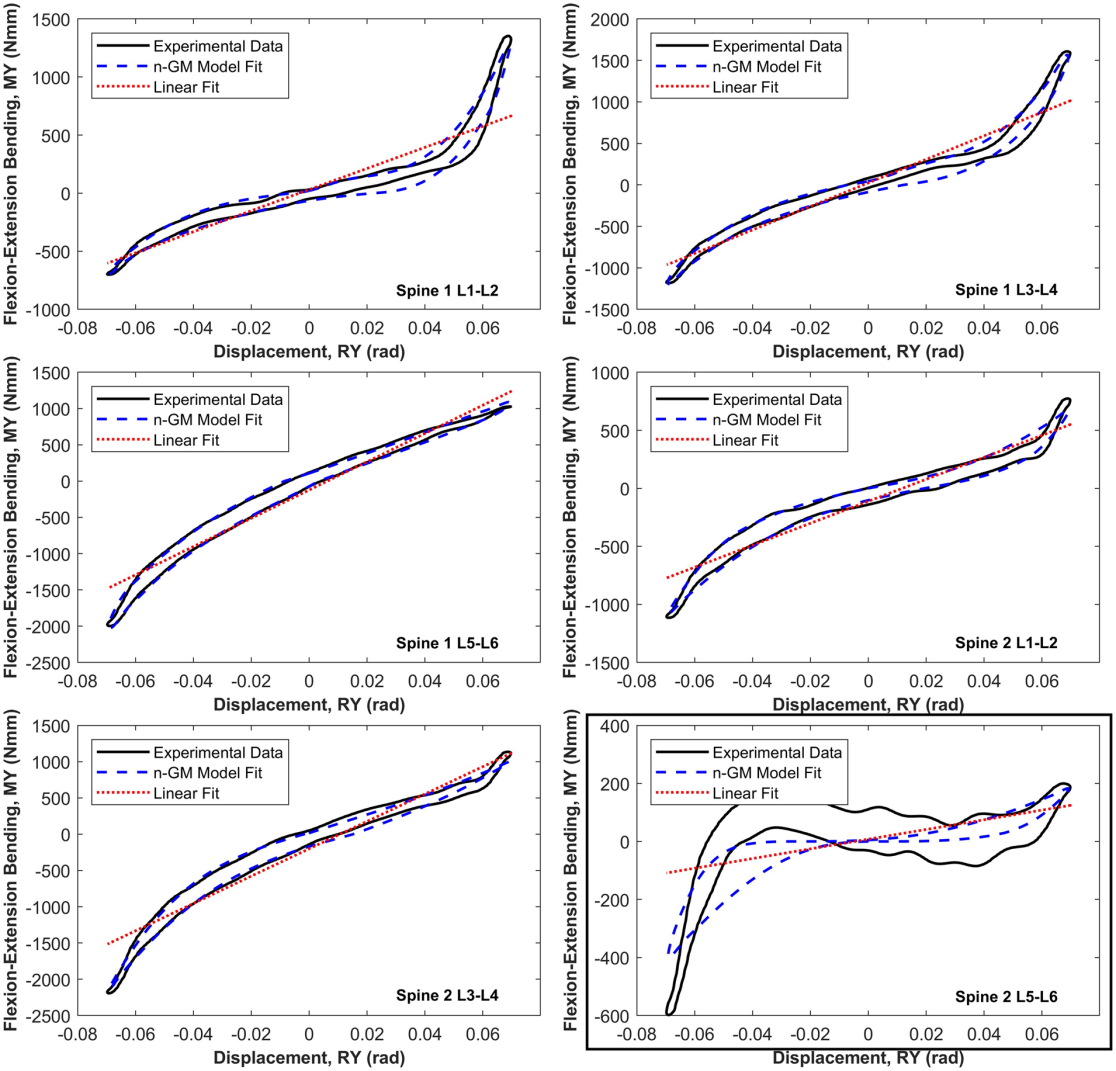
Resulting best fit of the first order Generalised Maxwell (n-GM) model to the flexion-extension (RY) principal element load-displacement behaviour for each of the six specimen tests. The linear model (LS) is also indicated for comparison. Specimen six, 2 L5L6, boxed in the figure, presented with abnormal behaviour.

Figure 5 illustrates the model fit in flexion-extension (RY), comparing the LS and asymmetric n-GM models. While the majority of specimens followed expected load-displacement trends, specimen 6 (L5–L6) exhibited an atypical response characterised by substantially lower loads and a negative gradient in the central region of the curve. This deviation is highlighted in Figure 5.

**Figure 6.**
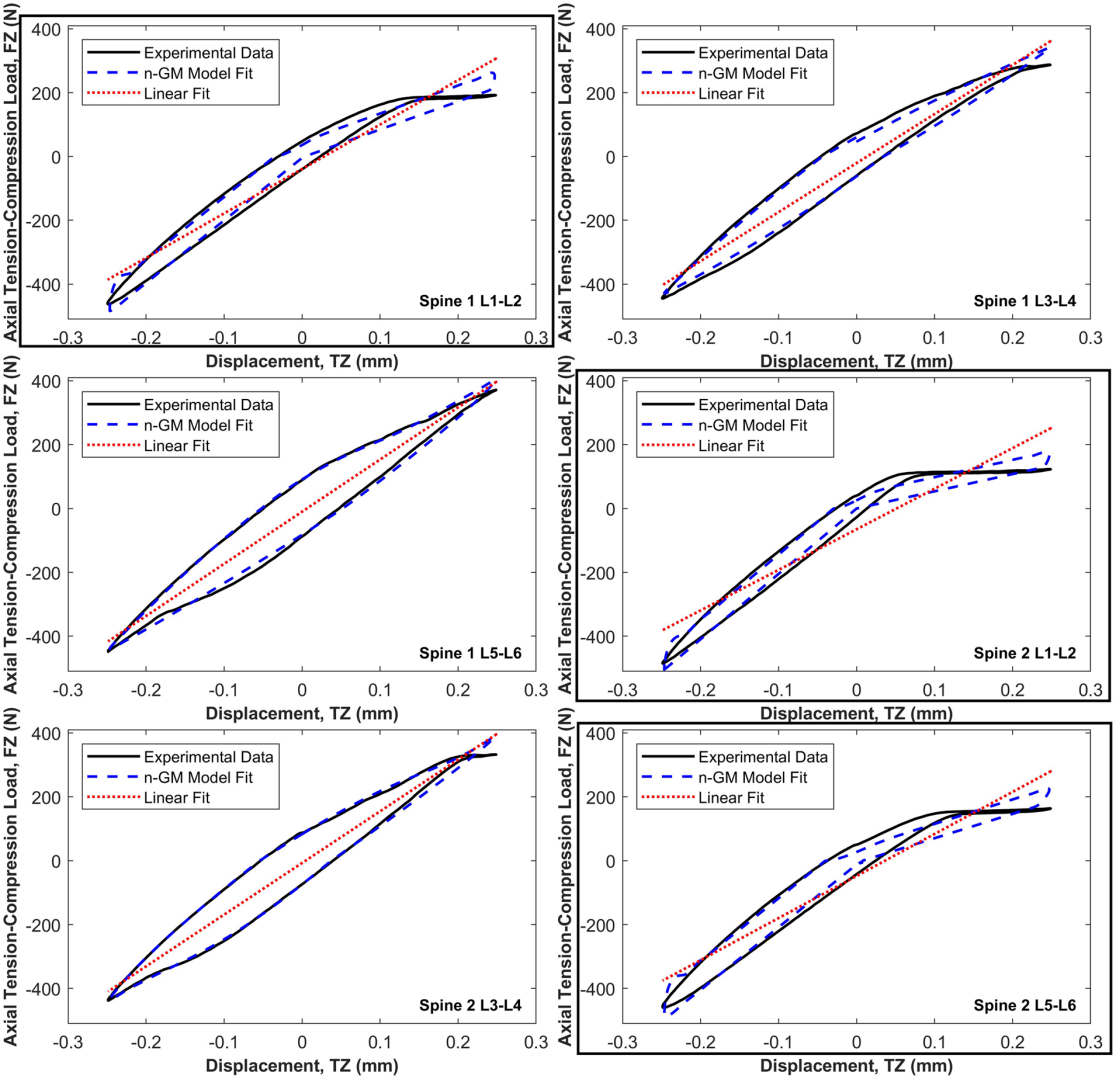
Resulting best fit of the first order Generalised Maxwell (n-GM) model to the axial compression-tension (TZ) principal element load-displacement behaviour for each of the six specimen tests. The linear model (LS) is also indicated for comparison. Specimens with a substantial horizontal step-region in the tensile portion are enclosed for emphasis.

Figure 6 shows the model fit in axial tension-compression (TZ). In three specimens, a plateau region was observed in the tensile portion of the load-displacement curve, resulting in reduced model fidelity. This behaviour introduced a visible transition in the fitted coefficients between positive and negative displacement and velocity, which is evident in the boxed regions of Figure 6.

The LS model is incapable of replicating hysteresis due to the lack of any viscous elements in the model. All nonlinear models (n-KV, n-GM, n-GK) reproduce experimental hysteresis within 10% (Table 2), with the exception of the n-GK model in mediolateral bending (RX) which replicates experimental hysteresis to within a mean of 17.4% (shaded in Table 2) across the sample size. In the majority of cases, the viscoelastic models result in an overestimation of the experimental hysteresis. There are only two cases where models underestimate experimental hysteresis, these are indicated by asterisks in Table 2.

**Table 2.** Mean energy dissipation in mJ per displacement cycle for the six principal elements. Data are presented for the experimental hysteresis area and the mean difference between the hysteresis exhibited by each nonlinear viscoelastic model configuration and the experimental hysteresis.

Model	Symmetric models								
	TXFX			TYFY			RZMZ		
	n-KV	n-GM	n-GK	n-KV	n-GM	n-GK	n-KV	n-GM	n-GK
Mean experimental hysteresis ( mJ )	100			23.4			45.8		
Mean % difference between model and experimental hysteresis	3.15 ↑	4.72 ↑	4.06 ↑	3.72 ↑	5.19 ↑	5.25 ↑	2.70 ↑	2.93 ↑	3.91 ↑
	Asymmetric models								
Model	TZFZ			RXMX			RYMY		
	n-KV	n-GM	n-GK	n-KV	n-GM	n-GK	n-KV	n-GM	n-GK
Mean experimental hysteresis ( mJ )	38.5			38.4			20.6		
Mean % difference between model and experimental hysteresis	4.21 ↑	5.66 ↑	2.35 ↑	7.86 ↑	0.32 ↑	17.4 ↑	3.21 ↑	6.07*↓	4.91*↓

Differences greater than 10% are shaded. Where the mean model hysteresis area is greater than the mean experimental hysteresis, this is indicated by an upwards pointing arrow, and where it is smaller, a downwards pointing arrow. An asterisk (*) indicates the only two cases where models underestimate experimental hysteresis.

The n-KV, n-GM and n-GK models consistently offer substantially improved quality of fit compared to LS in all principal directions across the entire sample, with symmetric n-GK models and asymmetric n-GM models providing greater than 60% decrease in normalised RMSE across all axes over the whole sample group compared to the commonly implemented LS model (Table 3).

**Table 3.** Table showing the RMSEs of the best fitting nonlinear model configurations normalised to the RMSEs of the linear model fit in each principal direction and for each specimen, nRMSE. Data are presented for the full load-displacement response except in axial compression-tension (TZFZ) where data are presented for just the compressive portion as well.

Specimen		TXFX	TYFY	RZMZ		TZFZ		RXMX	RYMY
						Full	Compression		
1 - L1L2	nRMSE symmetric n-GK model	0.42	0.46	0.15	nRMSE asymmetric n-GM model	0.44	0.20	0.21	0.27
2 - L3L4		0.62	0.83	0.15		0.45	0.14	0.30	0.32
3 - L5L6		0.32	0.26	0.20		0.28	0.12	0.20	0.16
4 - L1L2		0.20	0.22	0.15		0.37	0.18	0.31	0.25
5 - L3L4		0.48	0.21	0.15		0.30	0.04	0.33	0.25
6 - L5L6		0.23	0.43	0.19		0.41	0.18	0.35	0.10
Average		0.38	0.40	0.16		0.37	0.14	0.29	0.22

Solution coefficients from this study are available via an online data repository.^58^

Across all principal directions, the nonlinear viscoelastic models demonstrated varying degrees of fidelity in replicating experimental load-displacement behaviour. The symmetric n-GK model performed consistently well in shear and torsional directions, while the asymmetric n-GM model showed improved accuracy in bending and axial loading conditions, particularly in capturing hysteresis and nonlinear transitions. Notable specimen-specific deviations such as atypical responses in flexion-extension and axial tension highlight the impact of specimen variability in spine biomechanics. Although formal comparative analysis between spine levels and individual specimens was not conducted in this preliminary study, qualitative trends suggest that more complex model configurations offer improved quality of fit to experimental data. These findings support the validity of the modelling approach and lay the groundwork for future studies involving larger sample sizes and statistical comparisons.

## Discussion

This study presents a method for characterising IVD load response in the six principal directions using multi-element nonlinear viscoelastic models. It uniquely investigates multiple nonlinear viscoelastic model configurations for describing IVD response subject to six-axis cyclic displacements at low strain rates. Understanding IVD response to motion and loading is crucial to developing a full understanding of spinal behaviour and determining what load and displacement conditions may contribute to degeneration, injury and pain. The six principal directions, where load and displacement occur in the same direction, were evaluated in this study as they represent the governing stiffness matrix terms.

The linear method (LS) represents an over-simplification, neglecting nonlinearity, asymmetry, stiffening and hysteresis. While not facilitating, like the linear method, the formulation of a single neat 36-element stiffness matrix, the n-KV, n-GM and n-GK configurations account for all these key features and represent the elastic and energy dissipative characteristics. The linear elastic (LS) model is unable to replicate any nonlinearities or energy dissipation due to the lack of viscous elements in the model. The n-KV, n-GM and n-GK nonlinear viscoelastic models improve substantially on the quality of fit for all six principal directions across the sample group of six porcine lumbar ISD specimens when compared to the LS model, reducing error relative to experimental data by >60% for all principal directions (Table 3). Furthermore, the n-KV, n-GK and n-GM models can be modified to account for asymmetry in behaviour (Equations (1) and (2)), which is particularly evident in flexion-extension and mediolateral bending. This modification comes at the cost of extra characterising coefficients however and therefore can decrease the ease with which these coefficients can be reliably attributed to specific behavioural features.

The KV model response was generally characterised by roundness at extremes of motion (example in Figure 3a) and lacks the tapering seen at ROM extremes in experimental data, which is better replicated by the three-element models, n-GM and n-GK (example in Figure 3b). The additional spring element in n-GM and n-GK models, in general, offers a more accurate description of experimental load response, specifically improving model fidelity at the extremes of ROMs (Figure 3).

Analysis of the optimisation cost function value at solution indicated that the Generalised Maxwell (n-GM) model was best suited for characterisation of load response in flexion-extension (RY), mediolateral bending (RX) and axial compression-tension (TZ). Motions in which the fluid phase, the nucleus pulposus, dominates the response. The Generalised Kelvin (n-GK) model offered the best characterisation of load response in shear (TX, TY) and torsion (RZ), where the solid phase, the annulus fibrosus, dominates the response. A study by Tamaki and Panjabi investigated the suitability of a modified version of this model configuration – a linear Kelvin element in series with a nonlinear spring – for characterising spinal load response in flexion-extension and mediolateral bending.^29^ Their results showed that the model was effective for the simulation of viscoelastic behaviour of the isolated disc, however the study was limited to a single specimen.^29^ The results presented here have corroborated the conclusions from Tamaki and Panjabi^29^ and further extended the investigation into the use of nonlinear viscoelastic models for full characterisation of the six-axis load response of spinal specimens subject to cyclic loading by analysing behaviour from six specimens and across all six axes of behaviour.

The sixth specimen in the sample group, 6-L5L6, presented with an abnormal experimental load-displacement response in flexion-extension (RY) which was distinctly atypical (boxed in Figure 5). The response for this specimen was characterised by substantially lower peak loads and a negative gradient in the central region. For these reasons, viscoelastic models were not able to provide a high quality of fit to the experimental data, though the RMSE of the nonlinear viscoelastic model fit still improved on the RMSE of the LS model. It is unclear what caused this abnormal behaviour in this specimen as its load response in the remaining five axes was similar to other specimens. A potential cause could be a loosening of the specimen fixturing resulting in lower loads and an abnormal load response.

In axial compression-tension, typical specimen load response is nominally symmetric. However, the load-displacement response curves for three of the six specimens in this study displayed a pronounced horizontal step-region in the tensile portion of the load response (boxed in Figure 6). This is likely due to the applied axial preconditioning load of 400 N which induces a state of pre-compression in specimens. When axial displacements are then applied to the pre-compressed specimens, the positive (tensile) displacements act to release the axial preload, but as a result of specimen relaxation during the duration of the six-axis testing procedure, these displacements and their rate of application (0.1 mm/s) are not large enough to induce tensile forces in the specimen. This is affected by how much the axial compressive preload has decayed by the time the specimen is tested, which is influenced by the order in which the axes are tested. These step-regions affects the quality of fit obtained by the viscoelastic models across the full load-displacement response. For this reason, asymmetric viscoelastic models were used to characterise specimen load response in this axis. This allowed the quality of fit of the models to be quantified and compared for the compressive portion of the response separately to the full response, given that the natural mode in the spine in vivo is axial compression not tension. The normalised RMSE of the best-suited viscoelastic model improved on the LS model by 63% over the full load response and by 86% in axial compression, on average (Table 3).

Hysteresis observed in experimental data is due to the viscoelastic nature of tissues and is a measure of energy dissipated during motion. Without any viscous elements, the LS model exhibits no hysteresis and offers no information on dissipated energy. Therefore, insight into health of disc tissue and its ability to dissipate energy effectively, is omitted. While replication of hysteresis is an important feature in any model used to characterise IVD response, accurate matching of dissipated energy magnitude is not necessarily synonymous with the best fit quality. For example, in anterior-posterior shear for specimen 1-L1L2, the n-KV model has the highest normalised RMSE (nRMSE) of the nonlinear viscoelastic models, but provides the best replication of experimental dissipated energy, differing by only 3.4% from the hysteresis of the experimental data (Figure 3a). In contrast, the n-GK model, which offers the best fit (lowest normalised RMSE (nRMSE)), differs from experimental dissipated energy by 4.4% (Figure 3b). Good replication of dissipated energy is crucial to a model’s effective description of experimental data. However, it should be quoted and considered in conjunction with a quantifiable measure of error and fit quality such as RMSE.

The use of viscoelastic models for characterising experimental data from ISD specimens offers significant advantages over the traditional linear stiffness by describing all key features and facilitating in-depth analysis of IVD response. Furthermore, solution coefficients have recognisable physical meaning when taken in the context of biological specimens. Analysis of their relative magnitudes can be used to infer useful details regarding the load response of spine segments.

Linear stiffness offers insight into load bearing of the IVD. Larger values indicate greater linearity and greater load ranges in response to displacements. Cubic stiffness gives insight into strain stiffening and can help infer neutral zone width, a measure of spinal stability.^12^ Damping provides insight into energy dissipation, representative of IVD shock-absorbing characteristics and potentially the health of the nucleus pulposus.^54^

This method could be extended to more complex models with additional elements. However, the likely increase in quality of fit to experimental data comes with increased model complexity. This reduces the ease with which intuitive meaning can be attached to individual solution coefficients.

In many studies exploring the use of viscoelastic models for the description and characterisation of the creep and relaxation response of the IVD, linear viscoelastic models comprising linear spring and viscous damping elements have been proven to yield good quality of fit to experimental data, providing a beneficially representative description of data from in vitro tests.^26,33–35^ Nonlinear viscoelastic models increase the complexity, with parameters such as the time constant, making expressions dependant on strain. The suitability of such models for analyses such as creep/relaxation behaviour should be investigated to determine whether nonlinear viscoelastic models can offer improved outcomes for other applications in addition to cyclic loading scenarios. The viscoelastic response of spinal segments is rate-dependent and higher loading rates which are more representative of dynamic and impact-like activities would likely elicit different mechanical behaviour. However, previous studies have shown that spinal segments exhibit similar qualitative trends, such as nonlinearity, stiffening and hysteresis, across a range of loading frequencies^43,44,59,60^ suggesting that the modelling approach may remain applicable under more dynamic conditions.

Recent studies have demonstrated that relatively simple rheological models can effectively capture the viscoelastic behaviour of intervertebral discs under various loading conditions.^28,38,39,42,43,45,59^ Sciortino et al.^28^ showed that classical models such as the Standard Linear Solid (SLS) and Nutting’s power law could accurately describe creep behaviour and disc height reduction under sustained axial compression, with model parameters correlating to disc mechanical state. Similarly, Groth and Granata introduced a Standard Nonlinear Solid (SNS) model that improved upon the SLS by incorporating strain-dependent stiffness, enabling better prediction of cyclic modulus and load relaxation at low frequencies, though their study was limited to axial compression.^43^ Liu et al.^59^ further extended this approach by applying the Zhu–Wang–Tang nonlinear viscoelastic model to high strain rate axial loading conditions, demonstrating its ability to replicate stress–strain behaviour in damaged discs.

The present work focuses on establishing a methodology for fitting nonlinear viscoelastic models to experimental data across all six degrees of freedom using porcine lumbar motion segments. While previous research has largely concentrated on axial compression or simplified loading scenarios, our study introduces multi-axis modelling that fully captures viscoelastic response in shear, bending, torsion and axial directions. The findings suggest that nonlinear viscoelastic models, particularly those incorporating asymmetric damping and stiffness, can replicate complex load-displacement behaviour with high fidelity, even under low strain rate conditions. This has potential applications in the development of specimen-specific finite element models, prosthetic disc design and predictive tools for spinal biomechanics. This represents a novel contribution to the field, offering a more comprehensive basis for future modelling of spinal segment load response. Future work will expand on this methodology using a larger dataset, enabling statistical comparison and further validation across spinal levels and loading rates.

The purpose of this study is to introduce and motivate a new method for full characterisation of IVD load-displacement response at low strain rates. Several limitations should be acknowledged. First, the sample size was limited to six porcine lumbar spinal motion segments, which restricts the statistical power and generalisability of the findings. Second, all testing was conducted at a single low strain rate, chosen to simulate slower movements; however, viscoelastic behaviour is known to be rate-dependent, and future studies should explore a broader range of loading rates. Third, while the study focused on validating the modelling methodology for individual segments, it did not include comparative analysis across different spinal levels or specimens. These limitations represent important avenues for future research, including expanded sample sizes, multi-rate testing and inter-segmental comparisons to further refine and validate the modelling approach.

The results presented in this paper are intended to demonstrate that substantial improvement in the characterisation of the 6 DOF response of the IVD to cyclic loading at low strain rates can be achieved by using nonlinear viscoelastic models to characterise the response instead of the commonly used linear elastic model.

## Conclusions

The method and results presented here demonstrate that nonlinear viscoelastic models comprising nonlinear sub-elements provide improved characterisation of the load-displacement response of porcine lumbar isolated spinal disc specimens subject to six-axis cyclic displacements at low strain rates. These improvements are demonstrated for a sample size of six porcine lumbar spinal segments. In this sample group, the symmetric nonlinear Generalised Kelvin (n-GK) model best represents load-displacement response in shear and axial torsion, while the asymmetric Generalised Maxwell (n-GM) model best represents load-displacement response in bending and axial tension-compression. These nonlinear viscoelastic models reduce RMSE by more than 60% compared to the commonly used linear spring (LS) model and replicate experimental hysteresis to within 6.5% across all axes. The presented method is motivated as a viable alternative to the traditional linear elastic model, addressing all key IVD behavioural characteristics and replicating IVD viscoelasticity that is evident in experimental data from in vitro tests.

## Supplemental Material

sj-docx-4-pih-10.1177_09544119251411015 – Supplemental material for Nonlinear viscoelastic models improve characterisation of 6 DOF intervertebral disc load response at low strain rates Supplemental material, sj-docx-4-pih-10.1177_09544119251411015 for Nonlinear viscoelastic models improve characterisation of 6 DOF intervertebral disc load response at low strain rates by Samantha Hayward, Patrick S. Keogh, Anthony W. Miles and Sabina Gheduzzi in Proceedings of the Institution of Mechanical Engineers, Part H: Journal of Engineering in Medicine

sj-docx-5-pih-10.1177_09544119251411015 – Supplemental material for Nonlinear viscoelastic models improve characterisation of 6 DOF intervertebral disc load response at low strain rates Supplemental material, sj-docx-5-pih-10.1177_09544119251411015 for Nonlinear viscoelastic models improve characterisation of 6 DOF intervertebral disc load response at low strain rates by Samantha Hayward, Patrick S. Keogh, Anthony W. Miles and Sabina Gheduzzi in Proceedings of the Institution of Mechanical Engineers, Part H: Journal of Engineering in Medicine

sj-jpg-1-pih-10.1177_09544119251411015 – Supplemental material for Nonlinear viscoelastic models improve characterisation of 6 DOF intervertebral disc load response at low strain rates Supplemental material, sj-jpg-1-pih-10.1177_09544119251411015 for Nonlinear viscoelastic models improve characterisation of 6 DOF intervertebral disc load response at low strain rates by Samantha Hayward, Patrick S. Keogh, Anthony W. Miles and Sabina Gheduzzi in Proceedings of the Institution of Mechanical Engineers, Part H: Journal of Engineering in Medicine

sj-jpg-2-pih-10.1177_09544119251411015 – Supplemental material for Nonlinear viscoelastic models improve characterisation of 6 DOF intervertebral disc load response at low strain rates Supplemental material, sj-jpg-2-pih-10.1177_09544119251411015 for Nonlinear viscoelastic models improve characterisation of 6 DOF intervertebral disc load response at low strain rates by Samantha Hayward, Patrick S. Keogh, Anthony W. Miles and Sabina Gheduzzi in Proceedings of the Institution of Mechanical Engineers, Part H: Journal of Engineering in Medicine

sj-jpg-3-pih-10.1177_09544119251411015 – Supplemental material for Nonlinear viscoelastic models improve characterisation of 6 DOF intervertebral disc load response at low strain rates Supplemental material, sj-jpg-3-pih-10.1177_09544119251411015 for Nonlinear viscoelastic models improve characterisation of 6 DOF intervertebral disc load response at low strain rates by Samantha Hayward, Patrick S. Keogh, Anthony W. Miles and Sabina Gheduzzi in Proceedings of the Institution of Mechanical Engineers, Part H: Journal of Engineering in Medicine
