# Supplementary material for: Nonlinear viscoelastic models improve characterisation of 6 DOF intervertebral disc load response at low strain rates
Source: Proc Inst Mech Eng H. 2026 Jan 25;240(2):139–52. doi: 10.1177/09544119251411015 (PMC12901689; doi:10.1177/09544119251411015)
Supplement: sj-docx-4-pih-10.1177_09544119251411015 – Supplemental material for Nonlinear viscoelastic models improve characterisation of 6 DOF intervertebral disc load response at low strain rates [file sj-docx-4-pih-10.1177_09544119251411015.docx]

Supplementary Figure 1: Resulting best fit of the first order Generalised Kelvin (n-GK) model to the anterior-posterior shear (TX) principal element load-displacement behaviour for each of the six specimen tests. The linear model (LS) is also indicated for comparison.

Supplementary Figure 2: Resulting best fit of the first order Generalised Kelvin (n-GK) model to the mediolateral shear (TY) principal element load-displacement behaviour for each of the six specimen tests. The linear model (LS) is also indicated for comparison.

Supplementary Figure 3: Resulting best fit of the first order Generalised Maxwell (n-GM) model to the mediolateral bending (RX) principal element load-displacement behaviour for each of the six specimen tests. The linear model (LS) is also indicated for comparison.
