# Supplementary material for: Nonlinear viscoelastic models improve characterisation of 6 DOF intervertebral disc load response at low strain rates
Source: Proc Inst Mech Eng H. 2026 Jan 25;240(2):139–52. doi: 10.1177/09544119251411015 (PMC12901689; doi:10.1177/09544119251411015)
Supplement: sj-docx-5-pih-10.1177_09544119251411015 – Supplemental material for Nonlinear viscoelastic models improve characterisation of 6 DOF intervertebral disc load response at low strain rates [file sj-docx-5-pih-10.1177_09544119251411015.docx]

|  | |  | **Symmetric n_GK Model** | | **Asymmetric n_GM Model** |
| --- | --- | --- | --- | --- | --- |
|  |  | Units | **Anterior-posterior shear** | **Mediolateral shear** | **Axial compression-extension** |
|  |  |  | Coefficients | Coefficients | Coefficients |
| **Viscoelastic model** | K_1_^1^ | *N/mm* | 27.187 | 96.470 | + 1605.6 |
|  |  |  |  |  | - 1966.3 |
|  | K_1_^3^ | *N/mm^3^* | 503.00 | 44.300 | + 0.025 |
|  |  |  |  |  | - 0.032 |
|  | K_2_^1^ | *N/mm* | 111.22 | 49.456 | + 4954.4 |
|  |  |  |  |  | - 757073 |
|  | K_2_^3^ | *N/mm^3^* | 25.473 | 0.0169 | + 286.64 |
|  |  |  |  |  | - 180.88 |
|  | c^1^ | *N/(mm/s)* | 33.469 | 1170.8 | + 692.52 |
|  |  |  |  |  | - 44.754 |
|  | c^3^ | *N/(mm/s)^3^* | 0.0000 | 0.0000 | + 0.0000 |
|  |  |  |  |  | - 0.0000 |

|  | |  | **Asymmetric n_GM Model** | | **Symmetric n_GK Model** |
| --- | --- | --- | --- | --- | --- |
|  |  | Units | **Mediolateral bending** | **Flexion-extension** | **Axial rotation** |
|  |  |  | Coefficients | Coefficients | Coefficients |
| **Viscoelastic model** | K_1_^1^ | *Nm/rad* | + 43.592 | + 0.0303 | 19.020 |
|  |  |  | - 32.644 | - 3.0226 |  |
|  | K_1_^3^ | *Nm/rad^3^* | + 0.0004 | + 10968 | 15962913.25 |
|  |  |  | - 35851 | - 42454 |  |
|  | K_2_^1^ | *Nm/rad* | + 12.226 | + 3.1608 | 52.780 |
|  |  |  | - 15.711 | - 4.1034 |  |
|  | K_2_^3^ | *Nm/rad^3^* | + 2351.1 | + 126640 | 33803.300 |
|  |  |  | - 4128.2 | - 5414.7 |  |
|  | c^1^ | *Nm/(rad/s)* | + 0.0000 | + 2.9720 | 15.420 |
|  |  |  | - 16.112 | - 0.2933 |  |
|  | c^3^ | *Nm/(rad/s)^3^* | + 0.0821 | + 3601.1 | 18837 |
|  |  |  | - 53361 | - 55209 |  |
