# Supplementary figures and images for: Nonlinear viscoelastic models improve characterisation of 6 DOF intervertebral disc load response at low strain rates
Source: Proc Inst Mech Eng H. 2026 Jan 25;240(2):139–52. doi: 10.1177/09544119251411015 (PMC12901689; doi:10.1177/09544119251411015)

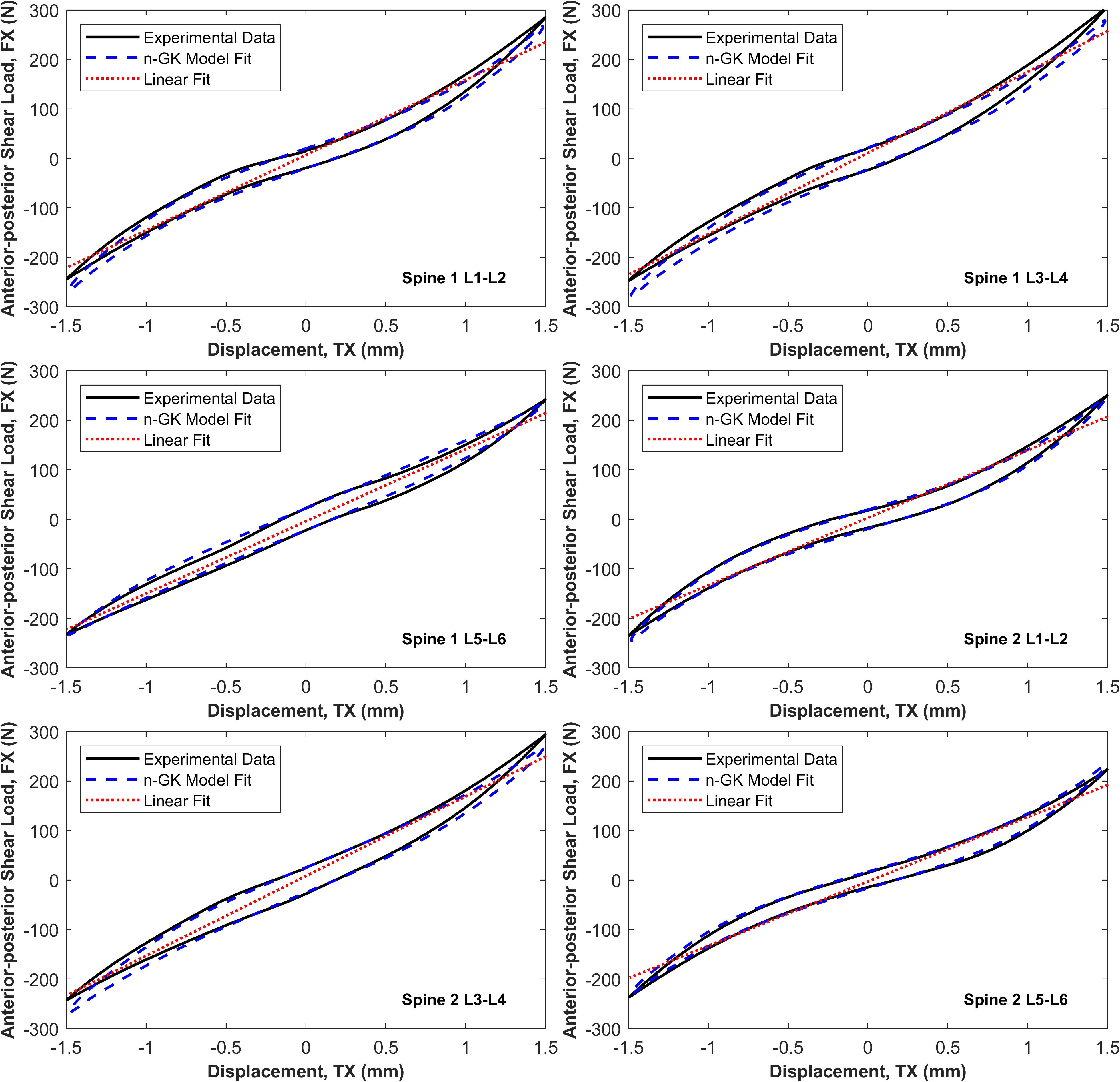

Supplement: sj-jpg-1-pih-10.1177_09544119251411015 – Supplemental material for Nonlinear viscoelastic models improve characterisation of 6 DOF intervertebral disc load response at low strain rates [file sj-jpg-1-pih-10.1177_09544119251411015.jpg]

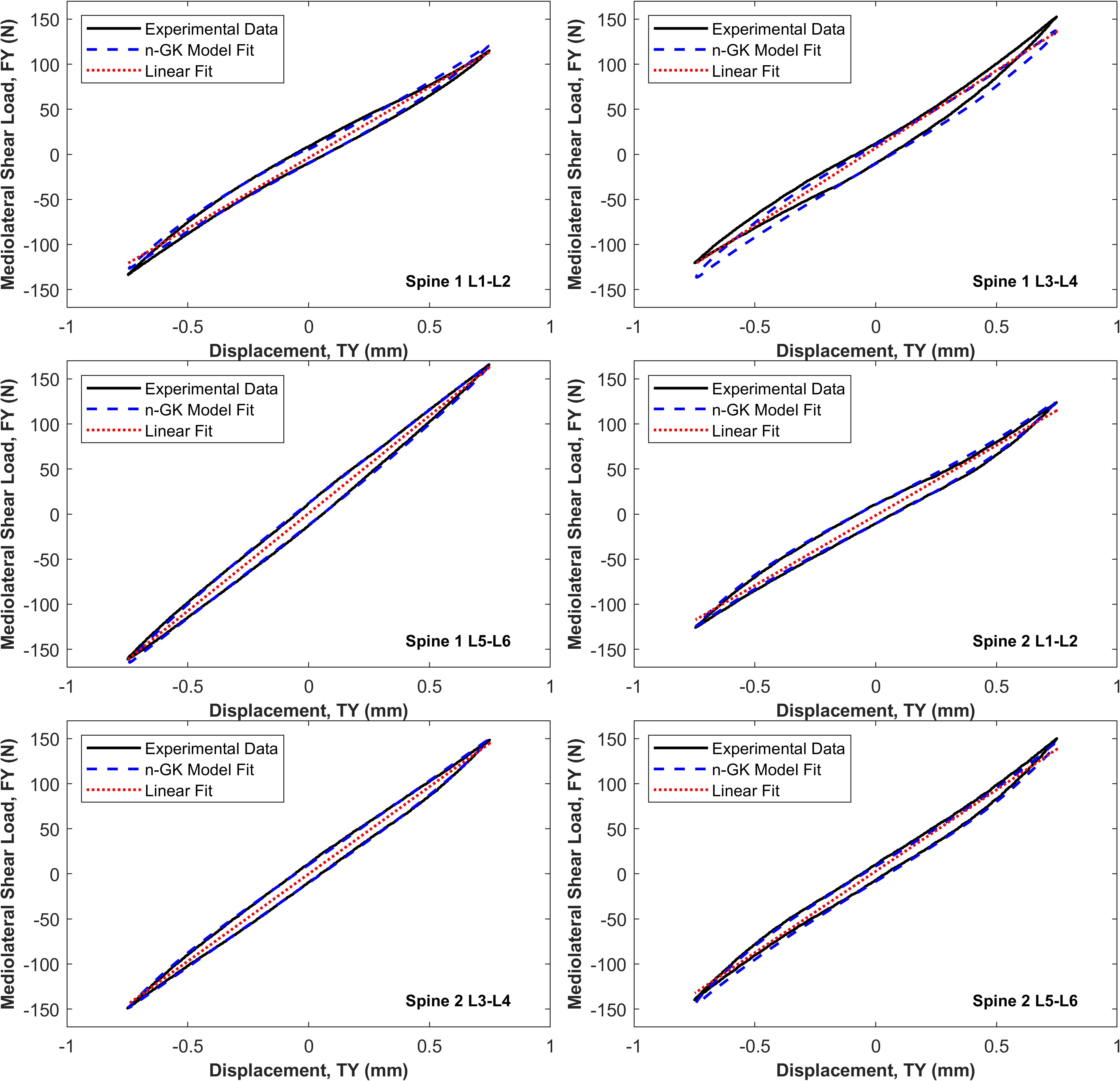

Supplement: sj-jpg-2-pih-10.1177_09544119251411015 – Supplemental material for Nonlinear viscoelastic models improve characterisation of 6 DOF intervertebral disc load response at low strain rates [file sj-jpg-2-pih-10.1177_09544119251411015.jpg]

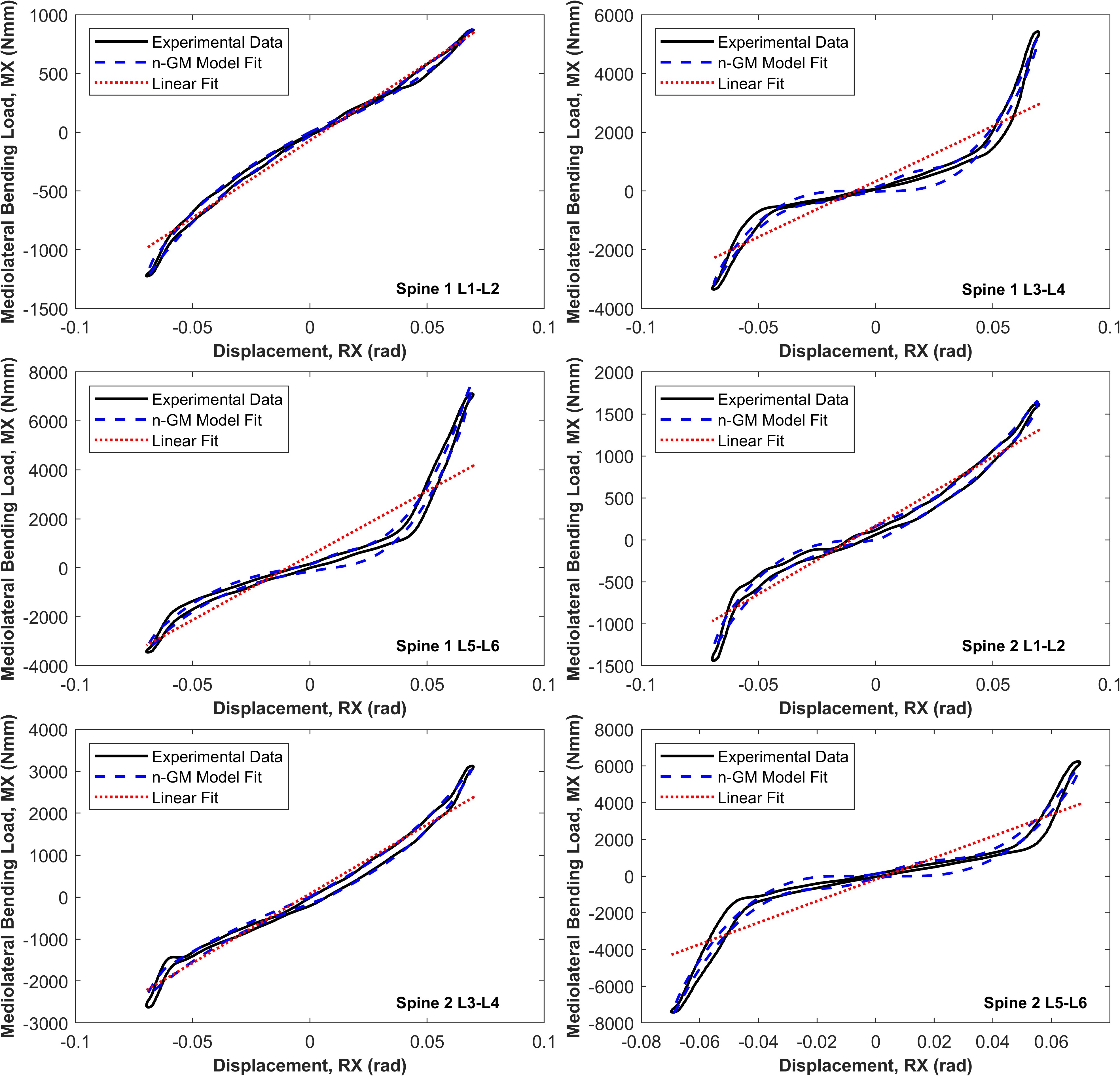

Supplement: sj-jpg-3-pih-10.1177_09544119251411015 – Supplemental material for Nonlinear viscoelastic models improve characterisation of 6 DOF intervertebral disc load response at low strain rates [file sj-jpg-3-pih-10.1177_09544119251411015.jpg]
